# Anti-allergic effect of a Korean traditional medicine, Biyeom-Tang on mast cells and allergic rhinitis

**DOI:** 10.1186/1472-6882-14-54

**Published:** 2014-02-12

**Authors:** Kyu-Tae Jeong, Sun-Gun Kim, Jiean Lee, Young Na Park, Hyo-Hyun Park, Na-Young Park, Keuk-Jun Kim, Hwadong Lee, Youn Ju Lee, Eunkyung Lee

**Affiliations:** 1 Research and Development Division, Korea Promotion Institute for Traditional Medicine Industry, Gyeongsan 712-260, Republic of Korea; 2 Center for Nutraceutical and Pharmaceutical Materials, Myongji University, Yongin 449-728, Republic of Korea; 3 Department of Clinical Pathology, Tae Kyeung College, Gyongsan 712-719, Republic of Korea; 4 School of Medicine, Catholic University of Daegu, Daegu 705-718, Republic of Korea

**Keywords:** Biyeom-Tang, Allergic rhinitis (AR), Bone-marrow derived mast cells (BMMC), Passive cutaneous anaphylaxis (PCA), Degranulation, Prostaglandin D_2_ (PGD_2_), Leukotriene C_4_ (LTC_4_)

## Abstract

**Background:**

Biyeom-Tang, a medicine prescribed by oriental clinics, has been used for the treatment of the allergic rhinitis (AR). In the present study, an ethanol extract of Biyeom-Tang (EBT) was investigated for anti-allergic properties on bone-marrow derived mast cells (BMMC) and *in vivo* models.

**Methods:**

The anti-allergic properties of EBT were evaluated by measuring β-Hex release and the production of prostaglandin D_2_ (PGD_2_) and leukotriene C_4_ (LTC_4_) on BMMC *in vitro* and PCA and OVA-induced AR models *in vivo*.

**Results:**

EBT strongly inhibited a degranulation reaction in a dose dependent manner with an IC_50_ value of 35.6 μg/ml. In addition, the generation of PGD_2_ and LTC_4_ was inhibited in BMMC in a concentration-dependent manner with IC_50_ values of 7.0 μg/ml and 10.9 μg/ml, respectively. When administrated orally, EBT ameliorated the mast cell-mediated PCA reaction. In the OVA-induced AR model, the increased levels of IgE were reduced by EBT. The levels of cytokines, such as IL-4, IL-5, IL-10, and IL-13 decreased in the splenocytes of EBT-treated mice. The histological analysis shows that the infiltration of inflammatory cells increased by OVA-sensitization was also reduced.

**Conclusions:**

Taken together, these results suggested that EBT has anti-allergic and anti-inflammatory effects *in vitro* and *in vivo* models.

## Background

Allergic rhinitis (AR) is a common chronic disease of nasal mucosa characterized by IgE-mediated allergic inflammation [1]. Although it is not life-threatening, AR can deteriorate the quality of life because of the clinical symptoms including sneezing, rhinorrhea, itching and nasal congestion. These symptoms are primarily induced by allergic mediators released from mast cells, basophils, eosinophils, lymphocytes, and epithelial cells. Among these cells, mast cells are one of the most important cells in the allergic response such as AR [2]. When activated, they release a number of biologically active molecules. Pharmacologically active mediators such as histamine, prostaglandins (PGs), leukotrienes (LTs) and cytokines have been known to play a major role in the pathophysiology of AR.

There are a number of effective therapeutic options, among which topical corticosteroids are recognized as the most important and the first line therapy for more severe and persistent forms of AR [3]. Available pharmacologic options for AR include anti-histamines, LT antagonists, and immunotherapy [4]. Since current available therapeutic agents have major adverse effects, further research on the medical management of AR is needed. Attention has focused on avoiding the adverse effects of medicines traditionally used to treat AR, which has stimulated the search for effective and safe alternatives.

The use of herbal medicines as an attractive approach for the treatment of various inflammatory disorders has been increasing. In an effort to find and demonstrate the efficacy of herbal formulae used in oriental clinics for the treatment of AR, Biyeom-Tang was evaluated for its anti-allergic activities. Biyeom-Tang, a Korean Traditional Medicine, composed of Xanthii Fructus (*Xanthium strumarium* Linne), Trichosanthis Semen (*Trichosanthe kirilowii*), Angelicae Dahuricae Radix, and Menthae Herba (*Mentha arvensis* Linne var. *piperascens* Malinvaud), has been used for the purpose of AR treatment in an oriental clinic. However, its anti-allergic properties and molecular mechanisms have not been investigated so far. The present study demonstrated the anti-allergic effects of an ethanol extract of Biyeom-Tang (EBT) using *in vitro* assays and *in vivo* models.

## Methods

### Plant materials

Herbs (*Xanthii* Fructus, *Trichosanthis* Semen, *Angelicae Dahuricae* Radix, and Menthae Herba) were purchased from Humanherb (Gyeongsan, Korea) and a voucher specimen has been deposited at the Korea Promotion Institute for Tradition Medicine Industry. All herbs were authenticated by Dr. H. Lee, a herbalist, in the Korea Promotion Institute for Traditional Medicine Industry. The herbs were mixed according to the ratio of combination (3:3:3:1.2), extracted with 70% ethanol at a ratio of 1:10 (w/v) and then refluxed for 24 h at 60°C. The extracted solution was filtered and the solvent evaporated under vacuum at 40°C (Eyela, Tokyo, Japan), before being freeze-dried to obtain a concentrated extract (11.70% yield).

### Preparation of bone marrow-derived mast cells (BMMC) and assay of β-hexosaminidase (β-Hex) release

Bone marrow cells from male BALB/c mice were cultured in 50% enriched medium (RPMI 1640 containing 2 mM L-glutamine, 0.1 mM nonessential amino acids, antibiotics and 10% fetal calf serum) and 20% pokeweed mitogen-stimulated spleen condition medium as a source of IL-3. After 3 weeks, BMMC were used for assays as the previously described procedure [5]. β-Hex was quantified by the spectrophotometric analysis of the hydrolysis of substrate (*p*-nitrophenyl-2-acetamido-2-deoxy-β-D-glucopyranoside, Sigma). Briefly, BMMC (1 × 10^6^ cells/mL) were pre-treated with EBT for 1 h and stimulated with KL (100 ng/mL) for 15 min. After harvesting supernatant, cells were lysed in the same volume of medium by freeze and thaw three times. Twenty five μL of BMMC lysate or supernatant was mixed with 50 μl of β-Hex substrate solution (1.3 mg/mL *p*-nitrophenyl-2-acetamido-2-deoxy-β-D-glucopyranoside in 100 mM sodium citrate, pH 4.5) in each well of 96-well plates and then incubated at 37°C for 60 min. The reaction was stopped by adding 175 μL of 0.2 M Glycin/NaOH (pH 10.7). The absorbance at 405 nm was measured in a microplate reader. The percentage of β-Hex released into the supernatant was calculated by the following formula: [S/(S + P)] × 100, where S and P are the β-Hex contents of supernatant and cell pellet, respectively.

### Measurement of PGD_2_ and LTC_4_

To measure cyclooxygenase (COX)-2-dependent PGD_2_ generation by EBT, BMMC suspended at a cell density of 1 × 10^6^ cells/mL in enriched medium were pre-incubated with aspirin (10 μg/mL) for 2 h in order to irreversibly inactivate pre-existing COX-1. After washing, BMMC were activated with *c-kit* ligand (KL, STEMCELL Technologies. Inc, Vancouver, BC, Canada), IL-10 (100 U/mL) and LPS (200 ng/mL) at 37°C for 8 h in the presence or absence of EBT and the supernatants were measured using an PGD_2_ assay kit (Cayman, Ann Arbor, MI, USA). For LTC_4_ determination, BMMC were pre-treated with EBT and stimulated with KL (100 ng/mL) for 15 min. Reactions were stopped by centrifugation at 120 g at 4°C for 5 min and the supernatants were measured using an LTC_4_ assay kit (Cayman).

### Passive cutaneous anaphylaxis (PCA)

ICR mice were obtained from Koatek (Seoul, Korea) and fed with laboratory feed (Purina, Seoul, Korea) and water *ad libitum*. Mice were acclimatized in a specific pathogen-free animal facility under conditions of 20–22°C, 40–60% relative humidity, and a 12 h/12 h (light/dark) cycle for at least for 7 d. For PCA, 80 ng of mouse anti-dinitrophenyl (DNP) IgE (Sigma) was intradermally injected into one ear of 7-week old male mice, followed 24 h later by oral administration of EBT (100 and 200 mg/kg) or fexofenadine-HCl, a histanime H1 receptor antagonist (Korea Pharma, Seoul, 50 mg/kg). One hour later, the mice were intravenously challenged with 60 mg of antigen (DNP-human serum albumin (HSA); Sigma) in 200 μl of PBS containing 1% (w/v) Evans blue. The mice were euthanized 1 h after treatment with the antigen, and their ears were removed and dissolved with 400 μl of formamide at 63°C overnight. The amount of dye extravasation was determined colorimetrically at 630 nm.

### Murine allergic rhinitis model and treatment

**Figure 1:**
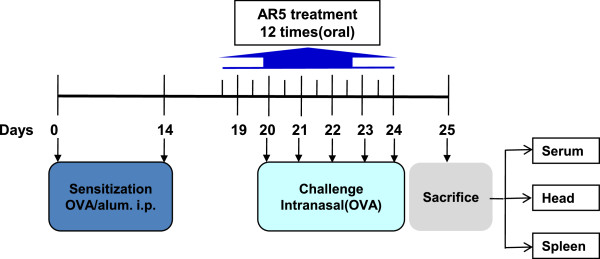
Sensitization and challenge protocol for the experimental murine AR model.

Six-week old female BALB/c mice (16–20 g) were sensitized by an intraperitoneal (i.p.) administration on days 0 and 14 with 20 μg/mL of OVA in PBS mixed with an equal volume of alum (1 mg) as an adjuvant in a total volume of 200 μL. The mice were challenged intranasally (i.n.) on days 20 to 24 with 100 μg OVA (5 μL in PBS) into each nostril. The EBT (50, 100, 200 mg/kg), or dexamethasone (Dex, 1 mg/kg) was per orally administered 12 times every 12 h from 1 day before the first challenge to the last challenge (Figure 1). Mice care and experimental procedures were performed under the approval from the animal care committee of Korea Promotion Institute for Traditional Medicine Industry (Approval No. KOTMIN-2012-007 for allergic rhinitis, KOTMIN-2013-013 for PCA).

### Measurement of IgE and cytokines

Blood was collected 18 h after the final challenge from mice via cardiac puncture and serum was obtained by centrifugation (1000 g for 10 min at 4°C) and stored -70°C until use. Total serum IgE was measured by using Mouse IgE ELISA kit (BD Biosciences, San Diego, CA, USA). The spleens were removed and single cell suspensions (4 × 10^6^ cells/mL) in a complete culture medium (RPMI 1640 containing 2 mM L–glutamine, 0.1 mM nonessential amino acids, 100 U/mL of penicillin, 100 μg/mL of streptomycin and 10% fetal calf serum) were cultured in 24-well plates in the presence of OVA (50 μg/mL). The supernatant was collected after a 72 h culture and the protein level of cytokines in the spleen culture supernatant was determined by ELISA according to the manufacturer’s protocol (R&D Systems, Minneapolis, MN, USA).

### Histological analysis of nasal mucosa tissue

The heads of mice were excised and fixed with 10% (v/v) formaldehyde. After fixation, the heads were decalcified in decalcifying solution-Lite (Sigma DO818) for 3 days. The specimens were dehydrated in various concentrations of ethanol and embedded in paraffin. The sections of the nasal mucosa were 3 μm thick and each section was stained with hematoxylin and eosin (H&E) and the periodic acid Schiff reagent (PAS).

### HPLC analysis

The chromatographic system was composed of a Waters 1525 Binary HPLC pump and a Waters 2998 Photodiode Array Detector (Waters, Corp., Milford, MA, USA). Detection and quantification were performed using Empower software. The separation was carried out on a Waters Sunfire C_18_ column (250 mm × 4.6 mm, 5 μm) at a column temperature of 40°C. The injection volume was 20 μL for a sample. The detection wavelength was 254 nm. The mobile phase consisted of Solvent A (0.05% aqueous TFA (v/v)) and Solvent B (acetonitrile) with gradient elution at the flow rate of 1 ml/min: 20% Solvent B at 0 min, 20% B at 2 min, 80% B at 46 min. Rosmarinic acid, imperatorin, and phellopterin were commercially available from Sigma and ChromaDex (Irvine, CA, USA).

### Statistical analysis

The data are expressed as mean ± SEM. One-way ANOVA was used to determine the statistical significance. The differences between groups in the AR model were evaluated by one-way ANOVA offered by Duncan’s multiple range tests. A value of p < 0.05 was considered significant.

## Results

### Effect of EBT on β-Hex release and eicosanoid (PGD_2_ and LTC_4_) production

**Figure 2:**
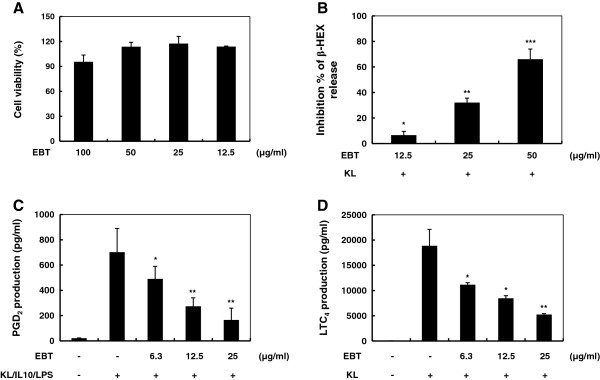
**Effect of EBT on cell viability, β-Hex release, LTC**_**4 **_**and PGD**_**2 **_**productions. (A)** BMMC were treated with different concentrations of EBT and the cell viability was measured. BMMC were pre-incubated with the indicated concentrations of EBT for 1 hr and stimulated with KL (100 ng/ml) for 15 min. Levels of **(B)** β-Hex and **(D)** LTC_4_ released into the supernatant were determined. **(C)** BMMC were pre-incubated with 10 μg/mL of aspirin for 2 h to abolish pre-existing COX-1 activity, followed by washing and then stimulation with KL/IL-10/LPS for 7 h. PGD_2_ released into the supernatant was quantified. *: P < 0.05, **: P < 0.01, and ***: P < 0.001 significantly different from AR mice as determined by a one-way ANOVA.

The effects of EBT concentration (50, 100, and 200 μg/ml) on cell viability were assessed by an MTS assay and there was no significant change in cell viability observed in the response to these concentrations (Figure 2A). When mast cells are activated through IgE-dependent or IgE-independent ways, they release preformed mediators from their granules and produce newly synthesized eicosanoids, chemokines and cytokines [6,7]. Degranulation was monitored by determining the release of β-Hex because histamine release by activated mast cells parallels the release of β-Hex. As shown in Figure 2B, pre-treatment with EBT resulted in a dose-dependent inhibition of β-Hex release, with an IC_50_ value of 35.6 μg/ml. Eicosanoids such as PGD_2_ and LTC_4_ secreted from activated mast cells have long been implicated in the etiology and manifestation of inflammation and allergic diseases [8]. To assess COX-2 dependent PGD_2_ generation, mast cells were pre-treated with aspirin to abolish any pre-existing COX-1 activity, and stimulated with the KL/IL-10/LPS. The PGD_2_ production was dose-dependently inhibited by EBT, with an IC_50_ value of 7.0 μg/ml (Figure 2C). We also assessed the inhibitory activity of EBT on LTC_4_ production and showed that EBT consistently inhibited LTC_4_ generation in a dose-dependent manner, with an IC_50_ value of 10.9 μg/ml (Figure 2D). These results suggested that EBT has COX-2/5-LOX dual inhibitory activities.

### Effect of EBT on PCA

**Figure 3:**
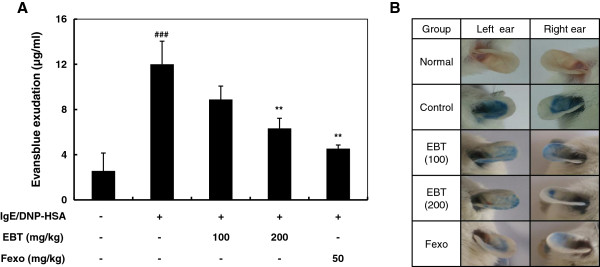
**Effect of EBT on systemic anaphylaxis and IgE/antigen-induced PCA reaction. (A)** Mice were given an intraperitoneal injection of compound 48/80 before EBT was orally administered. **(B)** EBT was administered orally 1 h prior to the challenge with antigen. Each amount of dye was extracted and measured as described in Materials and methods. The data were expressed as the mean ± SEM (n = 6). ###: P < 0.001 significantly different from the IgE/antigen unsensitized mice mice. *: P < 0.01, **: P < 0.05 significantly different from the IgE/antigen sensitized mice as determined by a one-way ANOVA.

A local extravasation was induced by a local injection of IgE followed by an antigen challenge. We examined the anti-allergic activity of EBT by using the PCA. An oral administration of EBT 1 h before injection of antigen inhibited the mast cell-mediated PCA reaction in a dose-dependent manner (23% at 100 mg/kg and 46% at 200 mg/kg), indicating that EBT exerts anti-allergic effects through the inhibition of mast cell degranulation (Figure 3).

### Effect of EBT on IgE and cytokine levels in the OVA-induced mice

**Figure 4:**
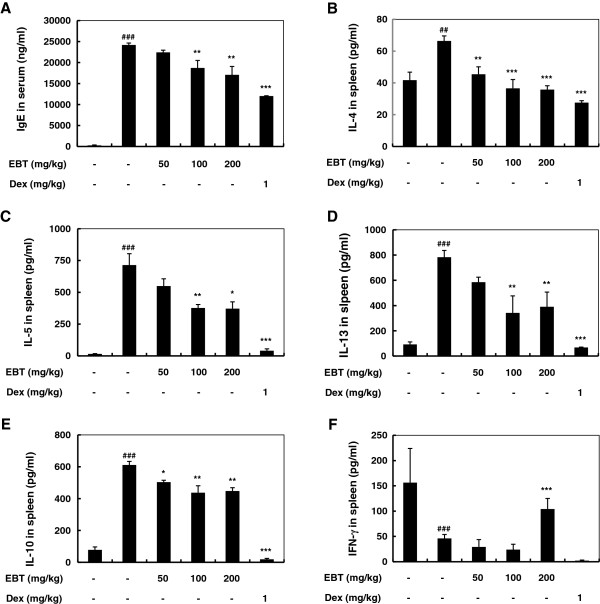
**Effect of EBT on the serum IgE level and cytokine response of splenocytes. (A)** Eighteen hours after the final challenge, blood was collected by cardiac puncture to measure the serum IgE. **(B-F)** The splenocytes were isolated and cultured in medium with OVA (50 μg/ml). Supernatants were collected after 72 h. The protein levels of IL-4, IL-5, IL-10, and IL-13 were determined by ELISA. The data were expressed as the mean ± SEM (n = 6). ###: P < 0.001 significantly different from the OVA-uninduced mice. *: P < 0.05, **: P < 0.01, and ***: P < 0.001 significantly different from AR mice as determined by a one-way ANOVA.

To determine the effect of EBT on the allergic responses, we measured the level of serum IgE in the OVA-induced mice. Figure 4A shows that the level of total IgE from serum was significantly up-regulated in OVA-induced mice when compared to the normal mice. However, treatment with EBT at all concentrations tested led to a decrease in the serum level of total IgE, indicating that EBT modulated the Th1/Th2 levels in OVA-induced AR mice. We also evaluated the effect of EBT on T-cell cytokine secretion by splenocyte cultures to determine its possible effects on the T-cell response. Figure 4B-E shows that the IL-4, IL-5, IL-10, and IL-13 levels were lower in cultures from the EBT-treated group than in those from the OVA-induced group, showing suppression of the Th2 response. IFN-γ secretion by splenocytes from the EBT-treated mice increased significantly in the EBT-treated mice compared to that of cultures from the OVA-induced mice (Figure 4F).

### Histological analysis of nasal mucosa tissue

**Figure 5:**
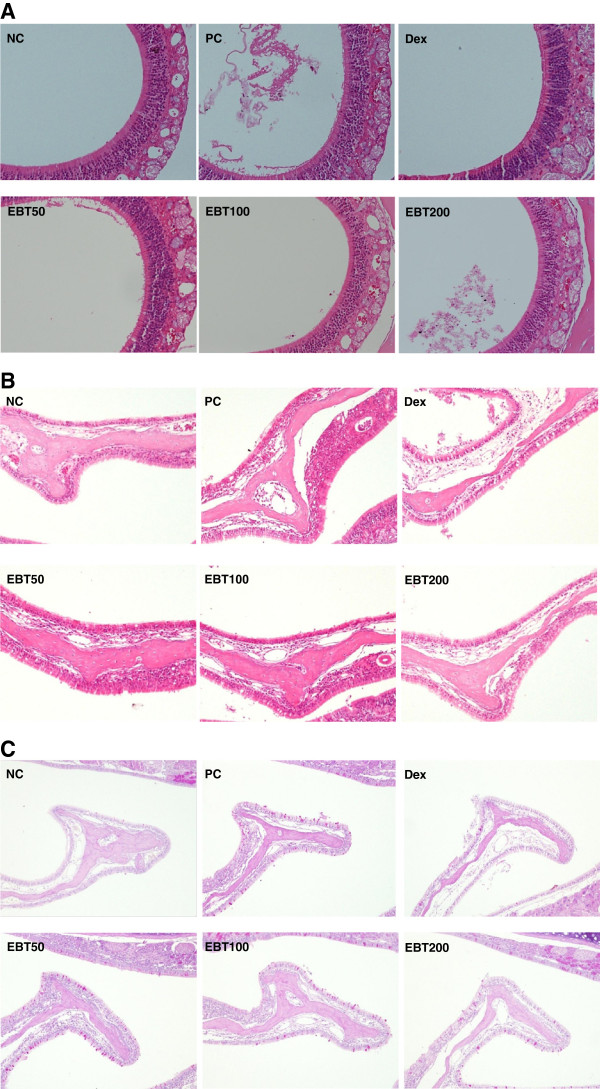
**Histological analysis of nasal mucosa tissue.** Eighteen hours after the OVA challenge, the nasal mucosa tissues were removed and histological analysis was conducted. The nasal cavities were decalcified and fixed. The sections of control (NC; PBS-induced, PC; OVA-induced) mice, and AR mice treated with EBT or Dex were stained with H&E (**A**, magnification x 200; **B**, magnification x 200) or PAS (**C**, magnification x 100).

The infiltration of leukocytes and mucus secretion are indexes of AR. We stained the nasal mucosa with H&E and PAS staining solution to examine the inhibitory effect of EBT on histological changes in the OVA-induced AR model. The inflammatory cells (such as mast cells and eosinophils) in the nasal mucosa of OVA-induced mice increased compared to those in control mice. A marked reduction in the infiltration of inflammatory cells was observed in the mucosa from mice that were treated with EBT (Figure 5A). The mucous membrane lining the nostril is shown in Figure 5B. In the OVA-induced mice, histological analysis showed the epithelial disruption and pre-treatment with EBT decreased the damage to the nasal epithelium, especially at a dose of 200 mg/kg. We also examined the effect of EBT on histological changes of the nasal mucosa by PAS staining. The mucus secretion in nasal mucosal tissue was increased in the AR mice compared with the control mice and EBT treatment reduced the mucus secretion (Figure 5C).

### HPLC analysis of EBT

**Figure 6:**
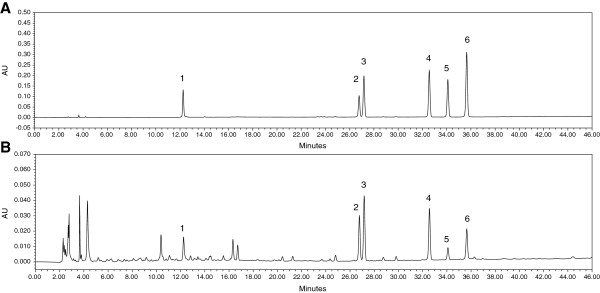
**HPLC chromatograms of EBT and marker compounds. (A)** The pattern of standard compounds. **(B)** The pattern of EBT (1, Rosmarinic acid; 2, Byakangelicol; 3, Oxypeucedanin; 4, Imperatorin; 5, Phellopterin; 6, Isoimperatorin).

The main compound profile of EBT was analyzed via the HPLC system. The identification of six compounds was based on the retention times and the UV spectrum in comparison with authentic standards at a wavelength of 254 nm. As shown in Figure 6, byakangelicol (2), oxypeucedanin (3), and isoimperatorin (6) were isolated and purified from *Angelicae dahuricae* Radix by the authors and the chemical structures were determined by the comparison of their NMR spectral data with authentic standards.

## Discussion

In this study, we demonstrated that Biyeom-Tang, a herbal formula used in an oriental clinic, had anti-allergic activities against degranulation and eicosanoid production in BMMC *in vitro* as well as in systemic anaphylaxis, PCA and OVA-induced AR model *in vivo*.

AR is an IgE-mediated inflammation characterized by Th2 type allergic reaction, and involves eosinophil infiltration, goblet cell hyperplasia, and mast cell accumulation in the nasal mucosa [4,9,10]. The early phase inflammatory response is primarily due to the release of mediators by mast cells. In the early allergic reaction, the binding of antigen-IgE results in the cross-linking of FeϵRI and subsequent degranulation of the mast cells, causing the release of active mediators such and histamine, PGs, LTs, and cytokines which produce symptoms of sneezing, itching and rhinorrhea. The late phase response is essentially a cellular event. Eosinophils are the predominant inflammatory cells that are activated by cytokines, chemokines, and histamine. Cytokines produced by Th2 cells play a pivotal role in the induction and maintenance of the allergic inflammatory cascade. The symptoms of the late allergic response are similar those of the early allergic response except that there is more-pronounced nasal congestion and mucus secretion.

Among effector cells, mast cells are one of the most important cells in allergic responses, such as AR [2]. Mast cells represent a major source of histamine, proteases, and other potent chemical mediators implicated in a wide variety of inflammatory and immunologic process. Activated mast cells degranulate and release preformed mediators or synthesize eicosanoid mediators from endogenous membrane arachidonic acid (AA) stores [6,7]. Among the preformed mediators, β-Hex, an acid hydrolase, is a marker of mast cell degranulation. Bioactive LTs, including LTB_4_ and cysteinyl LTs (cys-LTs, which include LTC_4_, LTD_4_, and LTE_4_) play major roles in several inflammatory diseases including asthma. LTC_4_ biosynthesis is initiated by the action of 5-lipoxygenase translocation to the nuclear membrane where it colocalizes with 5- lipoxygenase activating protein (FLAP), and uses AA released by cytosolic phospholipase A_2_ (cPLA_2_) as a substrate [11,12]. COX also converts AA, which is released from the plasma membrane via the action of cPLA_2_, into PGH_2_ and then into PGD_2_[13]. In this study, the inhibitory effect of EBT on KL-induced β-Hex release from BMMC was examined. As shown in Figure 2A, EBT inhibited β-Hex release in a dose dependent manner in BMMC. In addition, when BMMC were stimulated with KL or a combination of KL/IL-10/LPS with or without EBT, both the production of LTC_4_ and PGD_2_ was inhibited (Figure 2C and D). These results indicate that EBT suppresses the degranulation as well as the production of eicosanoids (PGD_2_ and LTC_4_) in mast cells.

Among the inflammatory mediators released from mast cells, histamine is one of the most potent vasoactive mediators implicated in the acute phase of immediate hypersensitivity [14]. Since PCA is one of the most important *in vivo* models of immediate hypersensitivity in local allergic reactions, PCA reaction was induced by the injection of IgE and antigen in this study. As shown in Figure 3, oral administration of EBT 1 h prior to challenge with antigen effectively reduced the PCA reaction in a dose dependent manner. These results clearly demonstrated that immediate-type allergic reactions are inhibited by EBT, indicating its role in the prevention or treatment of mast cell-mediated allergic diseases such as AR.

An imbalance between Th1 and Th2 responses lead to excessive Th1 cell or Th2 cells activation. Allergic diseases are characterized by a predominant Th2 response [15]. The allergic response in the nasal mucosa challenged with OVA in this study includes a late phase response characterized by recruitment of eosinophils, basophils, and T cells secreting Th2 cytokines [16]. In the present study, we successfully developed a mouse model of AR using OVA, showing higher serum total IgE, the infiltration of inflammatory cells, and Th2 cytokine secretion from splenoytes compared with the control group.

Th2 cytokines are known to play important roles in allergic disease. Because IFN-γ has been reported to inhibit the synthesis of IgE and the differentiation of precursor cells to Th2 cells, increased levels of IFN- γ have been used to explain the anti-allergic effects of therapeutic agents [17]. As shown in Figure 4B-F, we demonstrated that EBT reduced Th2 cytokine production (IL-4, IL-5, and IL-13) and increased Th1 cytokine (IFN- γ) production in cultured splenocytes, showing the anti-allergic effects of EBT. In addition, IL-10 is an inhibitory cytokine of inflammation and it was first identified as a Th2 cytokine and later revealed to be produced by Th1, Th2, Th17, and regulatory T cells [18,19]. In this study, IL-10 from splenocytes decreased, demonstrating the effect of EBT on the induction of regulatory T cells and the production of IL-10. These results indicate that EBT could regulate the balance of Th1/Th2 cytokine by inhibiting the development of allergic inflammation by shifting from a Th2 from a Th1 response in the OVA-induced AR mice.

Since IL-4 causes class switching in B cells to synthesize IgE production, the decreased IL-4 secretion from the cultured splenocytes results in reduced levels of serum IgE. TGF-β is known to inhibit inflammatory and immune responses through its anti-proliferative effects on T cells and macrophages whereas it plays a role in a signaling for fibroblast growth and activity in wound healing [20,21]. It was also suggested that active signaling is present in the nasal mucosa of AR and that this may play a role in the development of goblet cell hyperplasia in AR [22]. In this study, the level of serum TGF-β in OVA-induced AR mice slightly decreased, but not statistically after EBP treatment (data not shown). Therefore, the effect of EBT on the production of TGF-β in nasal mucosal tissue needs to be examined further.

There were increases of infiltrated inflammatory cells, disruption of epithelial cells, and secretion from mucous goblet cells in the nasal tissue, indicating five-time challenges with OVA in nostrils induced the allergic condition (Figure 5). These histopathological changes were alleviated by EBT treatment, suggesting that EBT is an effect Korean Traditional Medicine in the AR mouse model.

In our study, six compounds have been identified from EBT so far: one phenyl propanoid (rosmarinic acid) and five furanocoumarins (byakangelicol, oxypeudanin, imperatorin, phellopterin and isoimperatorin). A recent study has revealed that rosmarinic acid, a polyphenolic compound, showed anti-inflammatory activity in the treatment of allergic asthma [23]. The furanocoumarins also exert anti-inflammatory activities by inhibiting NO and PGE_2_ production, and among these compounds, imperatorin showed the most potent inhibitory activity [24-27]. Especially, imperatorin showed the anti-inflammatory effects by suppressing NF-κB and MAPK kinses in LPS-stimulated RAW 264.7 cells and a carrageenan-induced mouse paw edema model [27,28]. Furthermore, imperatorin alleviated the symptoms of AR in an OVA-induced mouse model [29]. In addition, imperatorin has been known to inhibit mucin secretion and so has possible use as a mucoregulator [30]. Although these compounds might be related to active components, other multiple compounds from EBT may have anti-inflammatory and anti-allergic activities. We are currently purifying other compounds from EBT to find biologically active compounds.

## Conclusion

In summary, EBT exerts anti-allergic effects in an OVA-induced AR mouse model by decreasing pharmacologically active mediators from mast cells *in vitro* and alleviates allergic rhinitis not only by suppression of Th2 cytokines and the serum IgE level but also by the decreased infiltration of inflammatory cells in the nasal mucosa. These multiple effects might synergize to reduce AR. However, the detailed molecular mechanisms involved and clarification of the active compounds in EBT need to be studied in further investigations.

## Competing interests

No competing financial interests exist.

## Authors’ contributions

KTJ, SGK and YNP carried out animal studies and the immunoassays. JL performed the HPLC analysis. NYP and HHP participated in the animal studies and performed the statistical studies. KJK and YJL participated in the histological analysis. HL provided the decoction of EBT. EL conceived of the study and drafted the manuscript. All authors read and approved the final manuscript.

## Pre-publication history

The pre-publication history for this paper can be accessed here:

http://www.biomedcentral.com/1472-6882/14/54/prepub
